# Flexible and Innovative Connectivity Solution to Support National Decentralized Infectious Diseases Point-of-Care Testing Programs in Primary Health Services: Descriptive Evaluation Study

**DOI:** 10.2196/46701

**Published:** 2023-09-01

**Authors:** Amit Saha, Kelly Andrewartha, Steven G Badman, Annie Tangey, Kirsty S Smith, Sergio Sandler, Stuart Ramsay, Wilton Braund, Stuart Manoj-Margison, Susan Matthews, Mark D S Shephard, Rebecca Guy, Louise Causer

**Affiliations:** 1 Kirby Institute University of New South Wales Sydney, NSW Australia; 2 Flinders University International Centre for Point-of-Care Testing Adelaide Australia; 3 Ngaanyatjarra Health Service Western Australia Australia; 4 Clinical Universe Adelaide Australia; 5 Flinders Medical Centre Adelaide Australia; 6 Australian Government Department of Health Canberra Australia

**Keywords:** infectious disease, diagnostics, POC testing, point-of-care, connectivity, digital health, disease surveillance, GeneXpert

## Abstract

**Background:**

Molecular point-of-care (POC) testing for *Chlamydia trachomatis* (CT), *Neisseria gonorrhoeae* (NG), and *Trichomonas vaginalis* (TV) has been available in regional and remote primary health services in Australia as part of a decentralized POC testing program since 2016 and for SARS-CoV-2 from 2020. As there was no suitable existing connectivity infrastructure to capture and deliver POC test results to a range of end users, a new system needed to be established.

**Objective:**

The aim of the study is to design, implement, and optimize a connectivity system to meet clinical management, analytical quality management, and public health surveillance needs.

**Methods:**

We used commercially available e-messaging technology coupled with adapted proprietary software to integrate a decentralized molecular POC testing platform (GeneXpert) in primary health services and interface with end-user databases. This connectivity infrastructure was designed to overcome key barriers to the implementation, integration, and monitoring of these large multijurisdictional infectious disease POC testing networks. Test result messages were tailored to meet end-user needs. Using centrally captured deidentified data, we evaluated the time to receipt of test results and completeness of accompanying demographic data.

**Results:**

From January 2016 to April 2020, we operationalized the system at 31 health services across 4 jurisdictions and integrated with 5 different patient management systems to support the real-time delivery of 29,356 CT/NG and TV test results to designated recipients (patient management system and local clinical and central program databases). In 2019, 12,105 CT/NG and TV results were delivered, and the median time to receipt of results was 3.2 (IQR 2.2-4.6) hours, inclusive of test runtime. From May 2020 to August 2022, we optimized the system to support rapid scale-up of SARS-CoV-2 testing (105 services; 6 jurisdictions; 71,823 tests) and additional sexually transmissible infection testing (16,232 tests), including the electronic disease-specific notifications to jurisdictional health departments and alerts for connectivity disruption and positive results. In 2022, 19,355 results were delivered with an overall median transmission time of 2.3 (IQR 1.4-3.1) hours, 2.2 (IQR 1.2-2.3) hours for SARS-CoV-2 (n=16,066), 3.0 (IQR 2.0-4.0) hours for CT/NG (n=1843), and 2.6 (IQR 1.5-3.8) hours for TV (n=1446). Demographic data (age, sex, and ethnicity) were completed for 99.5% of test results in 2022.

**Conclusions:**

This innovative connectivity system designed to meet end-user needs has proven to be sustainable, flexible, and scalable. It represents the first such system in Australia established independent of traditional pathology providers to support POC testing in geographically dispersed remote primary health services. The system has been optimized to deliver real-time test results and has proven critical for clinical, public health, and quality management. The system has significantly supported equitable access to rapid diagnostics for infectious diseases across Australia, and its design is suitable for onboarding other POC tests and testing platforms in the future.

## Introduction

Diagnostic technologies used at the point-of-care (POC) are changing the face of clinical management in settings where traditional laboratory-based diagnosis is difficult to access, unavailable, or result delivery is delayed. The individual and public health benefits of etiological diagnosis of infectious diseases by using POC testing are well-recognized including timely provision of curative treatment, minimizing the risk of sequelae, stopping onward transmission [1], and avoiding potential side effects as a result of unnecessary treatment [2]. POC testing can also contribute to antimicrobial stewardship by reducing the overuse of antibiotics and subsequent development of resistance and can be cost-saving [3]. However, translating research findings from trials and pilot projects and scaling up these benefits to a population level with POC testing integrated into routine clinical practice and supported as part of a decentralized diagnostic network can be challenging [4].

With the evolution of the decentralized diagnostics technology market, digital capabilities of test platforms, complementary software, and IT frameworks are becoming increasingly recognized as an essential component to support the integration and scale-up of decentralized testing [5]. The digital health data generated from newer technologies can be harnessed to guide patient care, monitor program implementation, guide analytical quality practices, and inform disease surveillance systems and the public health response [6]. Such test data can streamline the integration of POC testing at a health service by minimizing clinician workload, ensuring analytical quality, and enabling results to be available in real time to multiple end users to guide clinical management and link patients to effective care. Real-time connectivity is now considered one of the key criteria of the REASSURED (real-time connectivity, ease of specimen collection, affordable, sensitive, specific, user-friendly, rapid and robust, equipment-free or simple, environmentally friendly and deliverable to end-users) principles recognized by the World Health Organization pertaining to POC tests [7,8].

At the program level, digital health data can be used to monitor operator performance and quality of testing, alert program scientists to positive or invalid results, inform supply chain logistics, and maximize testing capacity and testing platform use across a large network of dispersed sites. At the regional or national level, these data contribute to disease surveillance, alert to disease outbreaks or emerging epidemics, and can inform and guide public health responses. New molecular-based testing platforms for infectious diseases such as the GeneXpert (Cepheid) generate digital data that are amenable to the timely capture of results and rapid transmission allowing real-time information for these critical purposes [9].

In Australia, most diagnostic testing is conducted in accredited pathology laboratories located in major urban and regional centers. POC testing has been available since 2011 for some chronic, noncommunicable diseases (ie, hemoglobin A_1c_ for diabetes) and acute conditions (ie, blood gases and white cell differential) [10,11]. Molecular POC testing has more recently become available for the sexually transmissible infections (STIs) including *Chlamydia trachomatis* (CT), *Neisseria gonorrhoeae* (NG), *and Trichomonas vaginalis* (TV) in several remote and regional primary health services through a national program. This program, known as TTANGO2 (Test, Treat and Go 2), commenced in 2016 and translated findings from a cluster randomized control trial [12] that demonstrated the acceptability [13,14], excellent operational performance [15], significant clinical impact [16], and cost-effectiveness [17] of POC testing in these settings compared to conventional laboratory-based testing. Molecular POC testing for these STIs is currently available at more than 60 regional and remote primary health services nationally and is supported by a comprehensive program of operator training and competency assessment, quality management, procurement, logistics for the supply of consumables and device maintenance, and on-demand troubleshooting and technical assistance. Molecular POC testing for SARS-CoV-2 was established and scaled up in May 2020, leveraging the experience and infrastructure established for the STI program [18]. The program is transitioning from the single SARS-CoV-2 assay to a multiplex respiratory panel assay and provides the capacity to test for SARS-CoV-2, influenza A and B, and respiratory syncytial virus (RSV) at the same time in one test.

In the absence of any existing framework in Australia to support such a geographically dispersed POC testing network in primary care settings, a connectivity system was designed and implemented to meet clinical, program, and public health needs including mandatory notification requirements. The connectivity system also needed to comply with Australian digital health regulatory guidelines and international standards. Here, we describe the design approach including challenges and solutions specific to the primary health service context in regional and remote Australia and the operational use of this novel connectivity system by considering uptake, effectiveness, and impact on programmatic implementation.

## Methods

### Connectivity System Goals

In collaboration with stakeholders [14], the following goals were identified: (1) integrate POC test processes into routine clinical practice and support clinical management; (2) securely capture, deliver, and store digital test outputs for program monitoring; (3) enable remote training, technical assistance, quality management, and device maintenance; and (4) enable positive alerts and mandatory notifications for public health surveillance. An overarching consideration was that achieving these goals should not increase clinicians’ workloads.

### Population and Setting

In regional and remote communities in Australia, health care services are delivered through primary care clinics that are staffed most often by 1-2 registered nurses, an Aboriginal and Torres Strait Islander health worker or practitioner, and sometimes a physician [19,20]. Turnover of staff is often high in remote locations [21]. These health services generally use electronic health records (EHRs) within a variety of digital patient management systems (PMSs) to record demographic data as well as consultation, procedures, testing, and diagnostic outcomes. Pathology results from centralized laboratories are usually returned electronically to the health service as a secure digital message (HL7 format; Clanwilliam) using an e-messenger service (HealthLink) so that the result can be easily reviewed and matched with the patient EHR to become part of the permanent patient record. These primary health services are often located many hundreds of kilometers away from centralized laboratories with specimen transport only once in a week; thus, results may take 7-10 days to be received at the clinic [22]. Most clinics share regional outsourced IT technical support to manage their routine clinical systems and IT-related issues.

### Public Health Surveillance for CT, NG, TV, and SARS-CoV-2

CT, NG, and SARS-CoV-2 infections are nationally notifiable, and TV is notifiable only in the Northern Territory. Notifications are regularly provided by pathology services and may be complemented by clinician-based reports [23,24]. Jurisdictional notifications are compiled nationally and reported annually for CT and NG [25,26]. During the SARS-CoV-2 pandemic, testing and case reporting were more frequent.

### National POC Testing Programs for STIs and SARS-CoV-2 in Australia

Molecular POC testing for STIs and SARS-CoV-2 is conducted by trained clinical staff using infection-specific cartridges with 4-module GeneXpert platforms located at the primary health service. In addition to providing the equipment for testing, the POC testing program provides comprehensive operator training to clinical staff (predominantly nurses and Aboriginal and Torres Strait Islander health workers and practitioners), quality management consisting of unique operator IDs, internal quality control and external quality assurance, and on-call technical assistance to health services as required (telephone and email helpdesk). The program governance, training, and quality management frameworks align closely with national POC testing guidelines [27].

### GeneXpert CT/NG, TV, and SARS-CoV-2 Tests and Platforms

The GeneXpert CT/NG test was approved by the Australian Therapeutic Goods Administration for the diagnosis of CT and NG infections in urogenital sites in 2013 [15], and the TV assay was approved in 2018 [28]. The GeneXpert SARS-CoV-2 test was approved by the Therapeutic Goods Administration in 2020. The CT/NG assay detects both CT and NG in 90 minutes using a single-patient specimen (endocervical or vaginal swab or urine); the TV assay result is available in 60 minutes using a separate cartridge. The single SARS-CoV-2 assay produces results within 45 minutes using an upper respiratory specimen (nasopharyngeal or midturbinate swab) [29,30]. All tests performed on the GeneXpert generate a digital qualitative result [31]. For CT/NG and TV, the successful qualitative results include “detected” or “not detected,” and for SARS-CoV-2, the results include “negative,” “presumptive positive,” or “positive.” Additional digital outputs include test date and time and specific analytes and cycle threshold values. Proprietary GeneXpert software (GeneXpert Dx) installed on a password-protected laptop accompanying the test device drives the testing process with results stored locally within an SQL (Microsoft) database.

### Software Applications and Databases

The proprietary GeneXpert Dx test software used in health services was not able to capture key patient information (patient identification number, first name, last name, date of birth, and contact details) to enable seamless result transmission to recipient databases in keeping with usual laboratory-based result delivery processes. We collaborated with specialist industry expertise (Clinical Universe) to adapt a middleware solution using proprietary software (ONDAS; Clinical Universe) and a complementary database (POC2Doc; Clinical Universe) to enable the generation, capture, transmission, and centralized storage of program POC test data. ONDAS is used by the clinical staff to generate the electronic POC test orders using mandatory patient identifiers. Patient and doctor lists with limited variables were generated from the health service PMS and configured within the ONDAS SQL database to prepopulate key patient information fields. This approach minimized the need for manual data entry and associated potential transcription errors and enabled the delivery of test results electronically to designated recipients (including PMS and other end-user databases). Details for new patients were able to be manually entered into ONDAS with the details retained in the ONDAS SQL database for future testing (stored locally). Updated patient and doctor lists were able to be installed as needed. This design ensured flexibility and adaptability to meet the variety of specific health service contexts encountered in regional and remote health services. The ONDAS middleware interfacing with the GeneXpert Dx software enabled the generation of a digital test result in HL7 format (HL7 tailored specifically to the requirements of each recipient) including with or without required patient information. These HL7 files are based on the Australian standard for HL7 and created by matching the generated ONDAS test order with the test results from the GeneXpert Dx software.

The POC2Doc database is located on a secure server at the Kirby Institute, University of New South Wales, Sydney, and was used to store deidentified result data for each test performed to completion, including patient, quality assurance, and training tests. Data included sample ID, patient ID, age at test, gender, ethnicity, test location, device and module number, cartridge lot number, operator name, date and time of test, date and time of receipt of the test result, and test result including analytes. Access to POC2Doc was available to end users using unique credentials via a secure web portal. This database was linked to visualization software (Tableau; Salesforce) for program monitoring and reporting to stakeholders.

We used HealthLink (Clanwilliam Company) to securely deliver the result messages (encrypted end-to-end) between individual decentralized testing platforms and designated recipients including local PMSs, centralized program database, and departments of health. We used LogMeIn (LogMeIn Inc) application to allow remote and secure laptop computer access as needed. These components are described in more detail in Textbox 1.

**Software and database descriptions of connectivity components.**

**ONDAS**

ONDAS (Clinical Universe) is a software product capable of interfacing with a range of point-of-care (POC) testing devices and manufacturers. The ONDAS user interface allows operators to enter key patient information (or quality assurance sample information) and generate an electronic test order, which is sent to the test devices for action. Once the test is complete, ONDAS captures the digital result output from the device; patient information is matched with the result, and a comprehensive result message is generated, which can be delivered to designated recipients (eg, electronic patient management systems and other data repositories) via a range of secure mechanisms. ONDAS is associated with a local SQL database where patient and results information can be securely stored. Digital output result messages (HL7, PDF, email, and others) are tailored to the requirements of each designated recipient. ONDAS conforms to all necessary testing and health data transmission standards, including HL7, UK National Health Service EDIFACT (Electronic Data Interchange for Administration, Commerce and Transport), ebXML, and POCT1-A.

**POC2Doc**

POC2Doc (Clinical Universe) is an SQL database designed to receive and store digital test outputs sent from multiple devices using ONDAS. POC2Doc provides a secure centralized repository of test data for program monitoring, quality management and operator competency, and local or jurisdictional reporting. Data housed in POC2Doc can be made visible and reportable to end users through secure web-portal access with assigned credentials.

**GxDx**

GeneXpert system software drives the testing process on the Gx platform.

**HealthLink**

HealthLink is an e-messaging service designed to securely transmit digital health data. HealthLink supports the transmission of encrypted digital result data from ONDAS to recipient databases (ie, local patient management systems and POC2Doc), which have a designated destination, HealthLink Electronic Documentation Interface.

**LogMeIn**

LogMeIn is a secure, comprehensive, and flexible-work provider software. It is a cloud-based remote work tool, which could connect to a remote desktop for file access from anywhere in the world.

**Figure 1:**
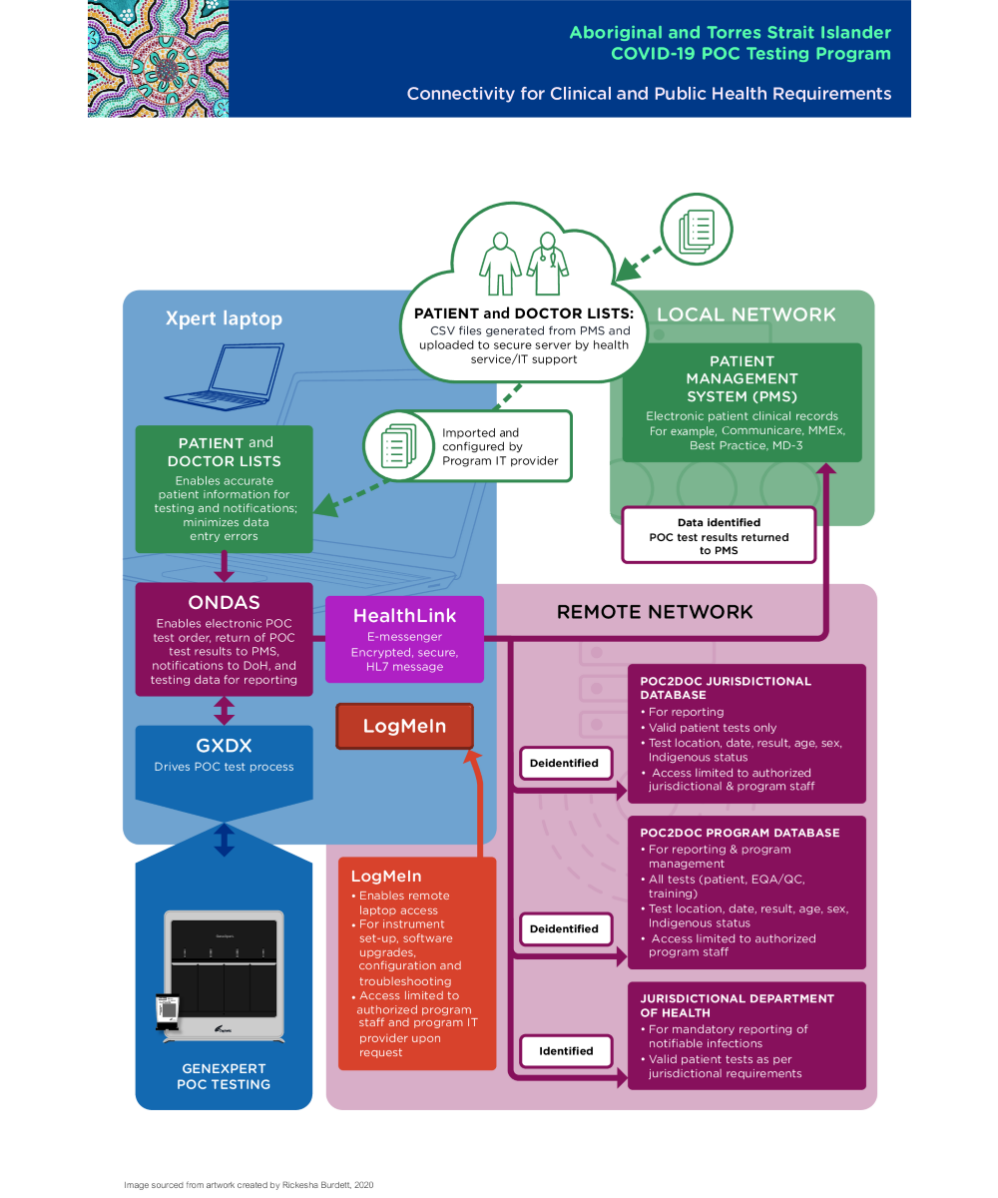
Schematic design of the connectivity system (2020). DoH: department of health; EQA: external quality assessment; GxDx: GeneXpert Dx; MD: medical director; MMEx: medical message exchange; PMS: patient management system; POC: point-of-care; QC: quality control.

All software applications were installed on the GeneXpert device laptop prior to field deployment or via remote installation. All subsequent system and IT configuration refinements were performed remotely. To transfer relevant data to several recipients, internet connections were established for each device either through USB 4G dongles that connect directly to the laptops or standalone Wi-Fi to 4G devices and on some occasions connected the laptops to the local services internet networks. Figure 1 provides a schematic design of the connectivity system using these applications and databases.

### Data Analysis

We conducted descriptive analyses using deidentified data captured in the centralized POC2Doc program database (located on a secure server at the Kirby Institute, University of New South Wales) to evaluate the number and type of tests conducted, time to receipt of results (time from the start of the test to time result received in POC2Doc), and completeness of patient demographics. Additional system and implementation information was obtained from program staff (personal communication) and routinely collected program logs.

### Ethics Approval

Ethics approvals for TTANGO2 were gained from the following institutional ethics committees: University of New South Wales Sydney, Flinders University, Western Australian Aboriginal Health Ethics Committee, Western Australian Country Health Service Research Ethics Committee, Kimberley Aboriginal Health Forum Research Sub-Committee, Far North Queensland Human Research Ethics Committee, Townsville Hospital and Health Service District Human Research Ethics Committee, Aboriginal Health Research Ethics Committee of South Australia, Central Australian Human Research Ethics Committee, Human Research Ethics Committee of the Northern Territory Department of Health, and Menzies School of Health Research. The Aboriginal and Torres Strait Islander COVID-19 POC testing program and TTANGO3 program are not research studies. Routinely collected deidentified program implementation data from both these programs were used to contribute to this evaluation and did not require specific ethics approval.

## Results

### Integrate POC Testing Into Clinical Practice

Between 2016 and 2020, we implemented the system in 31 regional and remote primary health services across 4 jurisdictions (Queensland, Northern Territory, Western Australia, and South Australia): 25 Aboriginal community–controlled health services and 6 health services managed by the jurisdictional government. The system interfaced with 5 different proprietary PMSs: communicare (n=15), medical message exchange (n=12), best practice (n=2), medical director (n=1), and patient information care system (n=1). The system was able to be tailored to the local context. Table 1 describes the characteristics of these health services and the degree of adoption of the system components.

**Figure 2:**
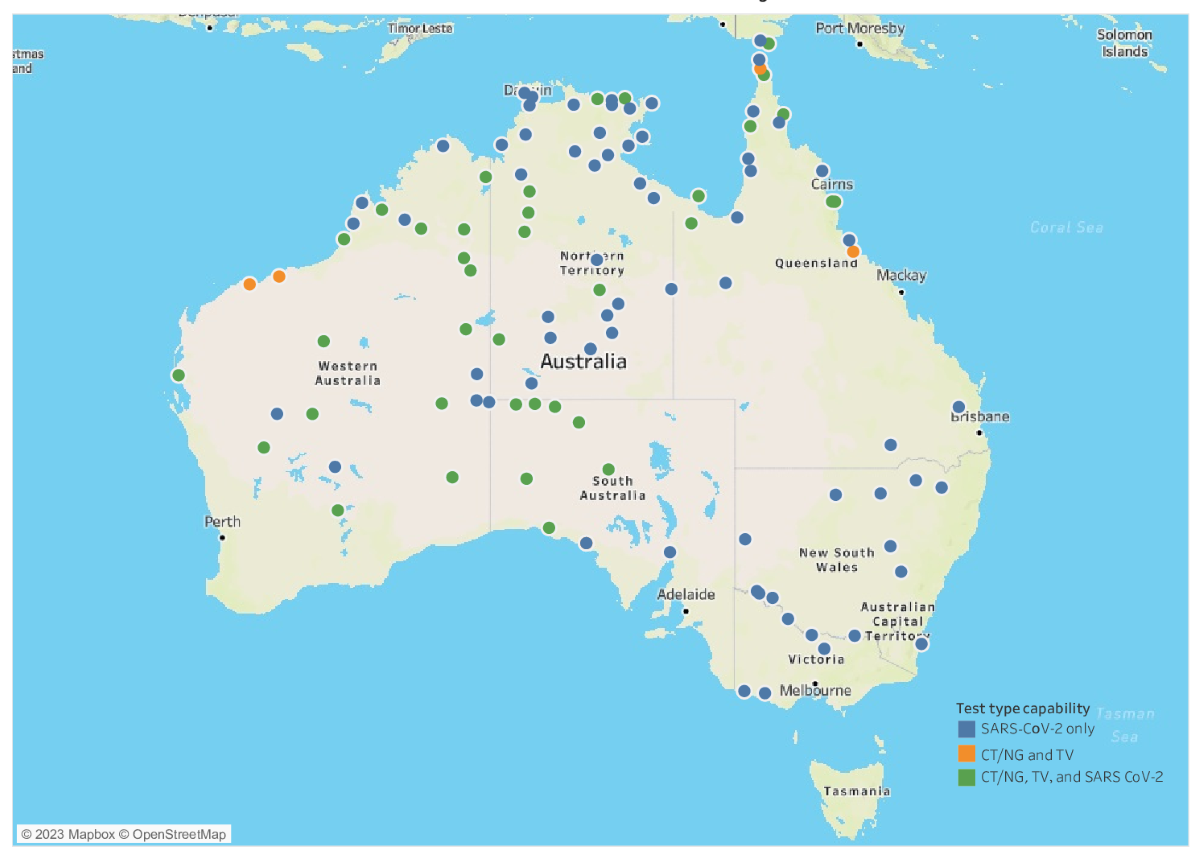
Decentralized CT/NG/TV and SARS-CoV-2 point-of-care testing networks (2022). CT: *Chlamydia trachomatis*; NG: *Neisseria gonorrhoeae*; TV: *Trichomonas vaginalis.*

All services had ONDAS installed on the device laptop; 29 services used patient and doctor lists. Electronic return of results for integration into the patient EHR was implemented at 25 sites. Overall, 6 sites were limited by government policy regarding the return of test results, which needed to be centralized rather than delivered to a local PMS; 2 sites (part of the same health service) declined the use of electronic test order generation and patient lists and elected results to be delivered to an existing local database for recording STI testing rather than into the PMS. Health services were encouraged to make a manual record or use a clinical item record in the individual patient EHR of the POC test performed and the result. Sites using ONDAS to generate test orders and patient lists experienced fewer data entry errors and made fewer requests for support from the helpdesk to safely action data entry errors (KA personal communication). The most frequent preanalytical errors reported from sites not using ONDAS test orders were incorrect entries of patient IDs. From May 2020 to August 2022, the system was scaled up to 105 primary care health services (Figure 2) across 6 jurisdictions to support SARS-CoV-2 testing.

In this program, ONDAS test orders and patient lists were implemented at all but 4 sites. Among these, 77 sites delivered results into the patient EHR. In total, 28 health services in 3 jurisdictions (mostly government-managed health services) required the adoption of a modified return of result process to a centralized pathology repository rather than the local PMS.

**Table 1:** POC^a^ testing program and site characteristics by program type.

Site characteristics			Program type^b^	
			STI,^c^ n	COVID-19, n
**Management**			31	105
	Jurisdictional government	7		37
	Community controlled	24		68
**Geographic location (ABS^d^)**				
	Very remote and remote	27		81
	Regional	4		24
**Jurisdiction**				
	Queensland	6		20
	Northern Territory	6		36
	South Australia	4		10
	Western Australia	15		22
	New South Wales	0		9
	Victoria	0		8
**PMS^e^ type**				
	Communicare	21		58
	MMEx^f^	6		9
	BP^g^	3		9
	MD-3^h^	1		5
	Others			21
**Test result recipients**				
	POC2Doc	31		105
	Local PMS	25		77
	Centralized results repository^i^	2		28
	Jurisdictional DoH^j^	0		105

^a^POC: point-of-care.

^b^STI program: January 2016 to April 2020; COVID program: May 2020 to August 2022 (includes 60 sites also doing STI POC testing).

^c^STI: sexually transmissible infection.

^d^ABS: Australian Bureau of Statistics.

^e^PMS: patient management system.

^f^MMEx: medical message exchange.

^g^BP: best practice.

^h^MD: medical director.

^i^Local, regional, or jurisdictional clinical results repository.

^j^DoH: department of health.

### Capture, Delivery, and Storage of Digital Test Outputs for Program Monitoring

From January 2016 to the end of August 2022, 117,411 test results (45,588 CT/NG and TV; 71,823 SARS-CoV-2) were received in the POC2Doc database. The combined (all test types) median result transmission time was 3.2 (IQR 2.2-4.6) hours in 2019 (CT/NG and TV only, n=12,105) decreasing to 2.3 (IQR 1.4-3.1) hours in 2022, 2.2 (IQR 1.2-2.3) hours for SARS-CoV-2 (n=16,066), 3.0 (IQR 2.0-4.0) hours for CT/NG (n=1843), and 2.6 (IQR 1.5-3.8) hours for TV (n=1446). This time included the test run time for each test (CT/NG: 90 min, TV: 60 min, SARS-CoV-2: 45 min). This improved transmission time was evident across all test types. Table 2 details the timeliness of results receipt by implementation year and test type.

In 2020, driven by the demand for real-time data to guide public health responses during the COVID-19 pandemic, several strategies were introduced to optimize results transmission. These included ONDAS email alerts to facilitate the rapid recognition of specific connectivity disruptions and allowed program staff to respond more quickly and rectify those amenable to intervention (such as loss of communications between host software and middleware, insufficient patient information entered, internet connections, and laptop or device failure). Some delays were beyond the control and remediation of the program staff including local or regional power outages or internet disruptions. Rapid recognition of issues allowed the program team to offer support to work around the disruption and provide results as needed to stakeholders manually until connectivity was restored. Operational experience with the system components and end-user setup has led to the program rigorously promoting type 1 connectivity setup (Table 3).

**Table 2:** Median time to receipt of result (time from start of test and result received into program POC2Doc database [CT/NG: January 2016 to August 2022, TV: April 2018 to August 2022, and SAR-CoV-2: May 2020 to August 2022]; median time includes the test run time [CT/NG: 90 min, TV: 60 min, and SARS-CoV-2: 45 min]) by test type and year.

Test type	Year						
	2016	2017	2018	2019	2020	2021	2022
CT^a^/NG^b^, n	2221	3721	5923	6885	4643	4662	1843
Time to results (hours), median (IQR)	5.1 (1.7-268.5)	3.7 (2.3-21.4)	3.4 (2.4-4.8)	3.3 (2.3-4.8)	3.9 (2.9-19.6)	2.8 (2.0-3.6)	3.0 (2.0-4.0)
TV^c^, n	N/A^d^	N/A	2440	5220	3506	3074	1446
Time to results (hours), median (IQR)	N/A	N/A	3.3 (2.1-4.3)	3.1 (2.0-4.3)	3.8 (2.6-13.2)	2.4 (1.6-3.5)	2.6 (1.5-3.8)
SARS-CoV-2, n	N/A	N/A	N/A	N/A	10,233	45,312	16,066
Time to results (hours), median (IQR)	N/A	N/A	N/A	N/A	2.4 (1.5-4.6)	1.4 (0.9-2.5)	2.2 (1.2-2.7)

^a^CT: *Chlamydia trachomatis*.

^b^NG: *Neisseria gonorrhoeae*.

^c^TV: *Trichomonas vaginalis*.

^d^N/A: not applicable.

**Table 3:** Connectivity type implemented across the POC^a^ testing network program.

Connectivity type	ONDAS test order generation or operator interface	Patient list used	POC2Doc (deidentified)	PMS^b^ (identified)	Other centralized repository^c^ (identified)	DoH^d^ (identified)
Type 1 (full connectivity)	✓	✓	✓	✓	✓	✓
Type 2 (limited connectivity)	✓		✓			
Type 3 (bespoke connectivity)			✓		✓	

^a^POC: point-of-care.

^b^PMS: patient management system.

^c^Local, regional, or jurisdictional clinical results repository.

^d^DoH: department of health.

Using the POC2Doc database and Tableau, an alert system was designed and enabled to highlight qualitative positive SARS-CoV-2 results allowing real-time result verifications by the program in support of quality control and device performance and enabling rapid public health responses to outbreaks.

The completeness of key test result variables (captured in POC2Doc) reflects the quality of data entry by the operator at the time of generating the test order either manually entered or prepopulated from patient lists. From 2018, patient testing information was enhanced to include age, sex, and ethnicity information captured as part of routine program data if the data were entered by the operator into the ONDAS middleware at the time of testing. In 2022, for health services using ONDAS test orders and patient lists (n=101), complete demographic data were available for 99.5% of test results (sex 99%, ethnicity 99.91%, and age 99.87%). The small proportion of missing data reflects tests performed outside the established connectivity pathway through inadvertent bypass of key data capture steps. Such process errors were addressed as needed through follow-up with operators.

### Support Remote Training, Technical Assistance, and Device Maintenance

LogMeIn applications installed on each laptop prior to field deployment enhanced operator training and allowed for rapid program support to investigate a range of operational and technical issues. These included data entry errors, invalid results, test results failing to reach designated recipients (POC2Doc, clinic EHR, or health departments), support operators when performing a POC test, review software, operating system status, and device settings, and perform software upgrades. Specialist technical support was engaged to resolve software, connectivity, or other IT issues beyond the capacity of program staff. These applications also provide information to program staff overseeing the high-level status of connectivity at individual sites.

### STI and SARS-CoV-2 Notifications for Public Health Surveillance

The connectivity system has the capability to deliver electronic test notifications (e-notifications) containing the POC test results and required patient information to jurisdictional departments of health for public health surveillance. The content and format of these notifications were designed to comply with the required data for mandatory infectious disease notifications. These notifications included patient, requesting doctor, and test result information and met HL7 standards (version 2.4).

e-Notifications were operationalized for the delivery of SARS-CoV-2 POC testing to all jurisdictional departments of health for integration into their infectious disease surveillance databases, including cross-border notifications where patients tested outside their jurisdiction of residence were notified to 2 jurisdictions in parallel. This process complements laboratory-based testing notifications and has been critical to track the COVID-19 epidemic in remote Aboriginal and Torres Strait Islander communities.

## Discussion

### Overview

This is the first description of an operational connectivity system designed to support large, multijurisdictional, decentralized infectious disease POC testing programs implemented at regional and remote primary health services in Australia. The system was specifically designed to overcome several challenges unique to the geographically remote locations of these primary health services, diverse IT environments, and multijurisdictional program context. The system demonstrated its capability to deliver real-time results with patient information to meet key clinical, program, and public health surveillance needs. The original system designed for the TTANGO2 STI POC testing program demonstrated its scalability and flexibility to accommodate a new assay in rapid response to a global pandemic delivered through the Aboriginal and Torres Strait Islander COVID-19 POC testing program [18]. Ongoing optimization of the system has substantially improved the time to results and quality of data collection with additional features including delivery of mandatory electronic notifications to jurisdictional departments of health for public health surveillance.

The specific design and functionality of this connectivity system responded directly to stakeholder-identified needs and primary care clinical pathways including the importance of streamlining clinical workflow, minimizing administrative and nonclinical tasks at the health service level, reducing the risk of data entry errors, ensuring reporting of notifiable infections, and maintaining public health surveillance with the increasing use of POC testing [29,32]. Importantly, all system components were established and able to be managed remotely, catering to the dispersed and geographic remoteness of many of the health services participating in the program.

At the jurisdictional and national level, reporting of notifiable infections and surveillance for antimicrobial resistance in the absence of routine laboratory-based diagnosis remains a high priority. The COVID-19 POC testing program operationalized and stressed the public health surveillance and notification functionality of the system with identified patient test results now being delivered to all 6 participating state and territory jurisdictional departments of health under the Public Health Act and Emergency Directives established as part of the national response to the COVID-19 pandemic. Additional features have been developed in response to stakeholder needs specific to COVID-19 including the generation of individual test result PDFs for provision to referral laboratories, an alert system to enable rapid program verification and public health response to any positive SARS-CoV-2 POC test results, and accessible web-based real-time data and reporting features for stakeholders.

This established connectivity system is supporting the current transition from the single SARS-CoV-2 assay to a multiplex respiratory assay (SARS-CoV-2, influenza A, influenza B, and RSV) and scale-up of CT/NG and TV testing across this network.

### Challenges

Several challenges were encountered during the implementation of the system, some unique to the remote Australian setting, while others are more broadly applicable to decentralized testing models. Most remote health services have limited IT capacity locally and share regional outsourced IT technical support to manage their routine clinical systems. Beyond some key initial advice as to the local IT structure and some assistance navigating firewalls and data flow pathways, ongoing support to the POC testing program was provided on an as-needed basis only. Instead, regular IT technical support was provided by our technical partners (Clinical Universe) and the program implementation team. Processes to maintain the connectivity system including software upgrades and technical assistance require ongoing IT support that must be sufficiently resourced.

Governance and regulations around third-party software providers and technologies challenged aspects of connectivity including the extent to which data could be integrated as part of routine collection. Although all health services had established internet connections, the reliability of the internet connection varied considerably, at times impacting results delivery and provision of technical assistance. Such disruptions lasted rarely more than 1 day with retrospective data available for delivery when the connection was re-established.

Despite operator training to navigate the laptop software, some have noted this to be a challenge to performing POC tests [33]. Ways to simplify, guide, or blend software interfaces should be explored to improve the user experience. Cost of connectivity establishment and ongoing support should be factored into program budgets. Cost-effectiveness modeling including some current connectivity costs does suggest that molecular POC testing for chlamydia, gonorrhea, and trichomoniasis for men and women is cost-effective in remote communities compared to standard laboratory testing [17].

While some distributed POC testing networks under the governance of established pathology providers exist (with POC testing devices in emergency departments and regional hospitals), our system is the first nationally operational system established specifically to meet the needs of regional and remote primary health services and range of clinical PMSs, thus enabling integration of this technology into routine care in these settings.

### Conclusions

In conclusion, this connectivity system has critically supported the implementation and integration of POC testing for STIs and SARS-CoV-2 at regional and remote primary health services across Australia while simultaneously ensuring quality assurance and public health surveillance needs are met. The system has supported more equitable access to rapid diagnostics for infectious diseases across Australia. Access to more timely diagnosis and treatment of these infections can lead to health benefits for the individual (reduced morbidity sequelae of infection) and community (reduced transmission and prevalence of infection). This system has been demonstrated to be technically scalable, amenable to onboarding new assays using the same GeneXpert testing platform, and able to accommodate a variety of expanded end users as required. The system is now supporting the incorporation of the GeneXpert multiplex SARS-CoV-2/influenza A/influenza B/RSV assay across the network [34] and is being used to support the new national hepatitis C virus POC testing program (since 2022), offering testing in other settings including drug treatment clinics, prisons, homelessness and mental health services, and some Aboriginal medical services [35]. This connectivity system can be further adapted to expand the scale of the network and suite of POC testing assays for other infectious diseases across Australia and could be suitable for similar settings internationally.

## Abbreviations

CT: *Chlamydia trachomatis*
EHR: electronic health record
NG: *Neisseria gonorrhoeae*
PMS: patient management system
POC: point-of-care
REASSURED: real-time connectivity, ease of specimen collection, affordable, sensitive, specific, user-friendly, rapid and robust, equipment-free or simple, environmentally friendly and deliverable to end-users
RSV: respiratory syncytial virus
STI: sexually transmissible infection
TTANGO: Test, Treat and Go
TV: *Trichomonas vaginalis*
WHO: World Health Organization
